# Tandem repeat expansions and copy number variations as risk factors and diagnostic tools for amyotrophic lateral sclerosis

**DOI:** 10.3389/fneur.2025.1522445

**Published:** 2025-02-12

**Authors:** Eleonora Sabetta, Davide Ferrari, Locatelli Massimo, Sulev Kõks

**Affiliations:** ^1^IRCCS Ospedale San Raffaele, Milan, Italy; ^2^SCVSA Department, University of Parma, Parma, Italy; ^3^Perron Institute for Neurological and Translational Science, Perth, WA, Australia; ^4^Centre for Molecular Medicine and Innovative Therapeutics, Murdoch University, Perth, WA, Australia

**Keywords:** ALS, STR, C9ORF72, ataxin, Huntington’s disease

## Abstract

Amyotrophic Lateral Sclerosis (ALS) is a neurodegenerative disorder leading to upper and lower motoneurons degeneration. Although several mechanisms potentially involved in disease development have been identified, its pathogenesis is not fully understood. From the patient side, ALS diagnosis, still based on clinical criteria, can be difficult and may take up to 1 year. More than 30 genes have been associated to genetically inherited ALS, among which four (C9ORF72, SOD1, TARDBP and FUS) would explain around 60–70% of cases. However, familial ALS represents only 5–10% of ALS cases while the remaining are sporadic, with genetics explaining 6–10% of such cases only. In this context, short tandem repeats (STRs) expansions, have recently been found in clinically diagnosed ALS patients. In this review, we discuss the recent discoveries on ALS associated STRs and their potential as biomarkers as well as prognosis and therapy targets.

## 1 Introduction

Amyotrophic Lateral Sclerosis (ALS) is a neurodegenerative disorder leading to upper and lower motoneurons degeneration (1), presenting a wide-ranging clinical phenotype (2) with an insidious onset and an unfavorable prognosis. Its pathogenesis is not fully understood, yet several mechanisms potentially involved in motor neuron degeneration have been identified, including neuronal hyperexcitability, mitochondrial dysfunction, oxidative stress, dysregulated vesicular transport, impaired DNA repair, and altered protein homeostasis (3). The majority of cases are considered sporadic (sALS) whereas 5–10% of ALS cases show a familial (fALS) inheritance (4). ALS diagnosis and classification is still based on clinical criteria (e.g., El Escorial criteria and Gold Coast criteria) and functional scales or staging systems like the ALS Functional Rating Scale-Revised (ALSFRS-R), the Milano-Torino (MiToS) functional staging and the King’s clinical staging, aiming at measuring disease progression. However, diagnosis can be difficult, the complete path can take up to 1 year and includes clinical, electrophysiological, and radiological investigations. Only recently, developed countries have begun offering genetic testing aimed at identifying mutations in specific genes known to cause ALS or increase the risk of developing the disease (5).

As research progresses, genetic testing and new reliable biomarkers would streamline the diagnostic process and/or in some cases predict different outcomes. Using whole genome sequencing (WGS) techniques will allow genomic profiling of patients and offer the opportunity to reveal the complexity of the ALS genetic landscape by identifying DNA sequences related to different phenotypes. This would represents a breakthrough especially for sALS, in which a clear genetic background is currently recognizable only in a minority of cases. In this context, Feldman et al. (4) recently proposed to replace the sALS and fALS classification with, respectively, non-genetically and genetically-confirmed ALS.

Investigation of ALS phenotypes related genes with the aim of reaching a diagnostic consensus based on underlying genetic sequences rather than clinical manifestations, could lead to improved screening programs and personalized therapies. In this context, short tandem repeats (STRs) expansions, often linked to neurodegenerative disorders (6), represent good candidates in the investigation of the molecular diagnosis of sALS.

Currently, more than 30 genes have been implicated in ALS (7), among which four (by decreased frequency of involvement: C9ORF72, SOD1, TARDBP and FUS) would explain around 60–70% of fALS cases and 6–10% of sALS (8). Interestingly, the pathogenic form of the chromosome 9 open reading frame 72 (C9ORF72) gene is a G4C2 hexanucleotide repeat expansion (HRE) in the intron 1 between the non-coding first exons 1a and 1b (9). Although C9ORF72 is currently the only STR expansion proven to cause ALS and frontotemporal spectrum disorder (FTD), different expanded STRs have been found in clinically diagnosed ALS patients and FTD cases (10). Such STR expansions were reported to be pathogenic for other neurodegenerative diseases like ATXN1 (spinal cerebellar ataxia type 1 (SCA1)), ATXN2 (SCA2), ATXN8 (SCA8) and HTT (Huntington’s disease) (6).

The STR research field is still in the initial stage and no clear evidence of the association between STRs and different ALS-related phenotypes or outcome prediction has been demonstrated. Nevertheless, the WGS results emerging from the current research setting studies contribute to the understanding of the genetic basis of this disease.

In this mini-review, we discuss the possible involvement of different STRs in the pathogenesis, diagnosis, prognosis and therapy of sALS.

### 1.1 A complex genetic architecture

In addition to the more than 30 genes associated with fALS (11, 12), heritability studies suggest a substantial genetic component (up to 60%) for sALS as well (13), yet only a few causal DNA sequences have been identified and SNP-based heritability is approximately 8.5% (10). This suggests that the pathological conditions of sALS are triggered by a complex genetic variation far from being understood. As an example, the most common known cause of fALS is the pathogenic C9ORF72 STR expansion (40–50%), which accounts only for approximately 7% of sALS (14). While known ALS disease genes account for the minority of sporadic cases, recent research highlights the potential role of noncoding structural variants and gene copy number variations in sALS susceptibility and phenotype modification. These variants are typically rare because they confer a significant increase in disease risk and a consequent negative selection pressure (15).

There is a growing interest in the oligogenic and polygenic inheritance of the disease risk (4), with some genes possibly exerting a pleiotropic effect on the phenotype (16). Indeed, some authors have shown the association between C9ORF72 variants with rapid cognitive decline, parkinsonism, or late-life neuropsychiatric symptoms (17). Moreover, a phenome-wide analysis highlights the pleiotropic nature of ALS by identifying 46 traits genetically correlated with ALS, including cognitive and behavioral traits (18). In this context, STRs expansions have already been linked to the ALS/frontotemporal spectrum disorder (ALS/FTD) development (e.g., C9ORF72 hexanucleotide G4C2 expansions) (19, 20) or identified as risk factors (e.g., CAG trinucleotide expansions in ATXN2) (4, 21), and could represent a valuable starting point to further investigate and clarify the genetic predisposition of sporadic cases as well.

Most of the STRs research studies have focused on their pathological role. However, given that over a third of the human genome includes repeat sequences (a million of which are STRs), scientists have investigated also their functional role. STRs appear to be involved in multiple biochemical processes: (1) they can affect the transcription of neighboring and distant genes by regulating chromatin structure and epigenetic markers; (2) they influence pre-mRNA splicing by forming complex higher-order structures, like G-quadruplexes and hairpins; (3) STRs located in the 5′ untranslated and coding regions impact mRNA translational efficiency by forming GC-rich stable RNA hairpins; (4) once translated into proteins, STRs become sites for protein–protein interactions (22, 23).

The pathological role of extended STRs could be related to an impairment of one of the above physiological roles, yet other disease-causing mechanisms might explain their broad involvement in neurodegenerative diseases. For instance, repetitive RNAs are prone to generate specific RNA/RNA and RNA/RNA-binding-proteins (RBP) interactions which may accumulate into aberrant nuclear aggregates, called RNA foci, often found in ALS patients (24). However, neurotoxicity may not arise from aggregates itself but rather by their ability to sequester key proteins *in vivo*, impairing their normal functions. In particular, RNA foci can sequester RBPs, key regulators of RNA metabolism. When RNA foci reach a critical level, they impair the mRNA metabolism, as RBPs are no longer available to perform their specific roles (25). In addition, an alternative pathological mechanism might arise from the formation of dipeptide repeats (DPRs) through a non-ATG-dependent translation of the extended STR. Several studies have demonstrated the toxicity of DPRs both *in vitro* and *in vivo* (26).

Understanding the biological mechanisms underlying the genetic variants associated to disease onset is also crucial for patients, as it directs diagnosis, potential personalized therapy and prognosis. STR expansions, such as those described in here, may be truly associated with disease risk; however, ALS appears as an extremely genetically complex disease. Large patient cohorts will be required to better inquire into the pathological significance of extended STR.

## 2 Discussion

### 2.1 The established role of C9ORF72 repeat expansions

Association between the C9ORF72 gene and ALS was observed in 2011 by two independent teams (9, 27) who identified a pathogenic G4C2 HRE in the intron 1 of the chromosome 9 open reading frame 72 gene, separating the non-coding first exons 1a and 1b. It is currently the most frequent genetic abnormality linked to ALS, with a dominant autosomal transmission bearing a 50% risk of developing the disease. However, penetrance might not be complete since 0.06% of the general population carry this expansion (12).

In addition, while C9ORF mutations are the most frequent cause of ALS in North American and European populations, this is not the case in other populations, such as in Asians, in which SOD1 mutations appear to be more prevalent (28).

Transcription of the C9ORF72 gene produces 3 variants depending on the alternative initiation and termination sites used (8). The first is a short transcript (C9-short) including the non-coding exon 1a and the coding exons 2–5 responsible for a 24 kDa protein. The second and third variants include the coding exons 2–11, leading to an identical 54 kDa protein (C9-long), preceded by either the non-coding exon 1b (variant 2) or the non-coding exon 1a (variant 3). Thus, all three variants share the exons 2–5 reading frame. RNA variants 4 and 5 were also detected when the alternative transcription start site in exon 1c was used. Remarkably, G2C4 HRE can be generated from the opposite strand as well and were detected in patient brains (29).

The mechanisms by which C9ORF72 HRE triggers or contributes to the neurodegenerative disease are not yet fully understood. Possible mechanisms based on published studies include: (1) HRE leading to impaired function of the gene product, (2) HRE leading to RNA toxicity effects and (3) the non ATG translation of HRE into toxic DPRs (8). Regarding the first mechanism, many studies have been carried out showing that the C9ORF72 protein has a fairly wide cellular distribution and is involved in many cellular processes, including autophagy regulation (30), inflammation (31), DNA repair (32) and cellular energy homeostasis (33). On the other hand, the second mechanism has been supported by studies showing that HRE alters transcription and induce genome instability (34) by forming secondary structures that accumulate in cell nuclei, sequestering various RBPs and resulting in nuclear RNA foci (35). Evidence supporting the third mechanism has also been found as DPRs sense or anti-sense have been detected in the neocortex, hippocampus, thalamus, and cerebellum of C9ORF72-associated ALS patients yet, DPRs were rare in the spinal cord (36). The fact that DPRs are more present in unaffected areas (cerebellum) and less detected in vulnerable regions (spinal cord) is controversial and led to the proposal hypothesis that DPRs might paradoxically exert a protective role (36).

Because of the strong association with fALS, several approaches (from anti-sense oligonucleotides (ASO) to viral vectors delivering technologies) have been set up to target C9ORF72 by gene therapy (37). Unfortunately, none of them have yet been translated into viable therapies for ALS patients (8).

### 2.2 HTT expansion and its controversial role

Huntington’s disease (HD) is an inherited neurological condition caused by a trinucleotide CAG repeat expansion (RE) within exon 1 of the Huntington gene (HTT). The HTT protein has neuroprotective functions: is essential for neuronal survival and is implicated in selective autophagy (38). The latter becomes relevant in neurodegenerative diseases, where protein aggregation and cellular stress are common. In this context the interaction between HTT and autophagy-related proteins like SQSTM1/p62 and ULK1 is crucial in maintaining cellular homeostasis under stress conditions (39).

Recently, the same pathogenic CAG RE (≥40 repeats) was found in patients with ALS (40). However, despite the large study cohort (2,442 FTD/ALS patients and 3,158 neurologically healthy individuals), only 3 patients (0.3%) were found to carry the HTT expansion compared to none in the control group. Because of these low percentages the study was questioned pointing out that the association between HTT expansions and ALS cases might simply arise by misdiagnosed cases of atypical HD or simply reflects the 0.03% HTT repeats prevalence among the general population (40, 41). Further studies, in which ALS diagnosis was confirmed after re-evaluation of medical records, showed similar results (10, 42). The percentages of ALS patients carrying HTT expansions slightly increases (1.5%) when considering HTT expansions in the reduced penetrance range (35–40 repeats) (42), and reaches 6.7% when intermediate lengths HTT repeats are considered (27–35 repeats) (43).

In light of the current studies, the role of HTT pathogenic expansions as causative of ALS is controversial. Nevertheless, shorter HTT expansion repeats have been found at a higher frequency in ALS patients suggesting that HD and ALS might be part of the same neurodegenerative disease landscape (44). Current data are still scarce and further cohorts are required to confirm the association and the pathogenic role of HTT REs within ALS.

### 2.3 Ataxin families’ expansion as a risk factor for ALS

Studies have shown that intermediate STR expansions in the spinocerebellar ataxia (SCA) genes, ATXN1 (SCA1) and ATXN2 (SCA2) are associated with ALS risk (45, 46). Yet, as observed for the HTT REs, the difference in frequency in ALS patients compared to healthy control subjects is narrow. Notably, a recent study involving 414 ALS patients and 713 control subjects from Scandinavian origin (42) showed no association between ALS and ATXN1 expansions whereas 1.7% carried >28 repeats in ATXN2 compared to 0.4% in healthy control. The ATXN2 gene codes for an RBP and regulator of stress granule assembly. CAG expansions of >34 repeats within ATXN2 are causative of SCA2 whereas shorter expansions (27–33 repeats) are now recognized as a risk factor for ALS. The first study, in 2010, compared 915 ALS patients to 980 controls and identified CAG RE (>26) to be enriched in the ALS population (5.5%) compared to controls (2.4%) (45). Subsequently, several groups have replicated such results, although the appropriate threshold cut-off varied from ≥29 to ≥31 (47–49).

At present, expansions of ≥31 repeats are associated with a markedly increased risk (odds ratio of 6.31) (50). These expansions are also linked to a more rapid disease progression and shorter survival (3). Additionally, the ATXN2 role in ALS is further supported by both its co-localization with known ALS proteins like TDP-43 and FUS in stress granules, and the observation that ATXN2 deficiency extends the lifespan of TDP-43-mutant mice (2). These findings collectively highlight the critical role of ATXN2 in ALS and the potential of targeting it for therapeutic interventions, as demonstrated by the beneficial effects of antisense oligonucleotide technology in preclinical models (2, 5). While our understanding of the precise role of ATXN2 in ALS is still evolving, its involvement underscores the intricate relationship between various neurodegenerative disorders.

## 3 Conclusion

The list of neurological disorders found to be caused by STR expansions is currently over 40, and is likely to grow (51). Although several hypotheses on STR expansions have been proposed over the years, its pathogenic mechanism is not yet fully understood. Recent studies, like those presented in this mini-review, showed that STR expansions may be truly associated with disease risk. However, so far, accurate identification of STR expansion regions has been challenging mainly due to technological and financial limitations. Until recently short-read sequencing was essentially the only option, yet it has several drawbacks that probably limited research progresses (52). In the last few years, the costly long-read sequencing has become more accessible and proved to be an effective method for identifying these targets, allowing for rapid and accurate genotyping (52). Moreover, alternative cost-effective approaches for the investigation of STR expansions have been tested with promising results (53, 54), leading to the increased identification of these mutations and further reducing the diagnostic gap.

The identification of REs as risk factors for ALS might have major clinical implications including improved understanding of the disease etiology and diagnostic advancements. Unraveling the reason by which STR expansions cause neurodegeneration may lead, in the near future, to effective novel therapies based on genetic approaches like CRISPR/Cas9 gene editing or antisense oligonucleotides.

Thus, genetic screening for REs could become a crucial component of ALS diagnostics whereas therapeutic strategies targeting the pathways involving REs may provide novel interventions for ALS. Nevertheless, future studies involving larger ALS patient cohorts will be required to elucidate the molecular mechanisms and potential therapeutic targets related to REs.
